# Severity identification for internet gaming disorder using heart rate variability reactivity for gaming cues: a deep learning approach

**DOI:** 10.3389/fpsyt.2023.1231045

**Published:** 2023-11-07

**Authors:** Sung Jun Hong, Deokjong Lee, Jinsick Park, Taekyung Kim, Young-Chul Jung, Young-Min Shon, In Young Kim

**Affiliations:** ^1^Biomedical Engineering Research Center, Samsung Medical Center, Seoul, Republic of Korea; ^2^Department of Psychiatry, Yongin Severance Hospital, Yonsei University College of Medicine, Yongin, Republic of Korea; ^3^Institute of Behavioral Sciences in Medicine, Yonsei University College of Medicine, Seoul, Republic of Korea; ^4^Division of Research Planning, Mental Health Research Institute, National Center for Mental Health, Seoul, Republic of Korea; ^5^Department of Medical Device Management and Research, Samsung Advanced Institute for Health Science and Technology, Sungkyunkwan University, Seoul, Republic of Korea; ^6^Department of Psychiatry, Yonsei University College of Medicine, Seoul, Republic of Korea; ^7^Institute for Innovation in Digital Healthcare, Yonsei University, Seoul, Republic of Korea; ^8^Department of Neurology, Samsung Medical Center, Sungkyunkwan University School of Medicine, Seoul, Republic of Korea; ^9^Department of Biomedical Engineering, Graduate School of Biomedical Science and Engineering, Hanyang University, Seoul, Republic of Korea

**Keywords:** deep learning model, heart rate variability, internet gaming disorder, behavioral addiction, addiction

## Abstract

**Background:**

The diminished executive control along with cue-reactivity has been suggested to play an important role in addiction. Hear rate variability (HRV), which is related to the autonomic nervous system, is a useful biomarker that can reflect cognitive-emotional responses to stimuli. In this study, Internet gaming disorder (IGD) subjects’ autonomic response to gaming-related cues was evaluated by measuring HRV changes in exposure to gaming situation. We investigated whether this HRV reactivity can significantly classify the categorical classification according to the severity of IGD.

**Methods:**

The present study included 70 subjects and classified them into 4 classes (normal, mild, moderate and severe) according to their IGD severity. We measured HRV for 5 min after the start of their preferred Internet game to reflect the autonomic response upon exposure to gaming. The neural parameters of deep learning model were trained using time-frequency parameters of HRV. Using the Class Activation Mapping (CAM) algorithm, we analyzed whether the deep learning model could predict the severity classification of IGD and which areas of the time-frequency series were mainly involved.

**Results:**

The trained deep learning model showed an accuracy of 95.10% and F-1 scores of 0.995 (normal), 0.994 (mild), 0.995 (moderate), and 0.999 (severe) for the four classes of IGD severity classification. As a result of checking the input of the deep learning model using the CAM algorithm, the high frequency (HF)-HRV was related to the severity classification of IGD. In the case of severe IGD, low frequency (LF)-HRV as well as HF-HRV were identified as regions of interest in the deep learning model.

**Conclusion:**

In a deep learning model using the time-frequency HRV data, a significant predictor of IGD severity classification was parasympathetic tone reactivity when exposed to gaming situations. The reactivity of the sympathetic tone for the gaming situation could predict only the severe group of IGD. This study suggests that the autonomic response to the game-related cues can reflect the addiction status to the game.

## Introduction

Internet gaming disorder (IGD) is defined as a psychiatric condition in which excessive game use cannot be properly controlled despite functional difficulties caused by excessive Internet game use (1). IGD is causing a lot of burden due to its high prevalence, and many researches are actively being conducted to provide evidence-based treatment for it (2, 3). IGD has been associated with a decline in executive function (4), and it has been found that people with IGD have difficulties controlling their emotions and impulses (5, 6). Although there are controversies, IGD is regarded as one of the addictive behaviors, and the diagnostic criteria for IGD has been presented in line with the existing diagnostic criteria for addiction (7, 8). Like addictive disorders, IGD has been associated with loss of control over addictive behavior and attentional bias to addiction-related cues (9, 10). Neuroimaging studies have reported that IGD represents the diminished prefrontal activity and the transition from the ventral to the dorsal axis in the striatum, similar to other addiction diseases (11, 12). However, it has been also reported that IGD is different from substance addiction in several clinical aspects and that the main symptoms of addiction such as tolerance and withdrawal are not conspicuous (13, 14). These somewhat confusing results suggest that more structured studies are needed on the disease characteristics and pathophysiology of IGD.

It has been suggested that addiction is accompanied by changes in incentive salience for addicted substance or behavior (15). Incentive salience is found to be reflected in the measurement of cue-reactivity for addiction-related cues (16). Studies on behavioral addictions such as IGD have also suggested an alteration of cue-reactivity (17). A disease model of behavioral addictions has proposed that executive control is specifically diminished for addictive behavior and stimulus-driven salience is strengthened for addiction-related cues (18). Theoretically, these speculations could be linked to the cognitive factors of IGD: preoccupation for gaming and decision-making deficit (8). However, most previous experimental studies did not explore executive control deficit of IGD in the context in which gaming-related cues were presented (19). Several previous neuroimaging studies studied IGD using the cue-reactivity task, but the alterations of brain activation in the reward network were not as prominent as expected (17). This was interpreted to be because the gaming-related cues (game-related words or pictures) used in previous studies differed in the degree of immersion from actual game play.

Alterations of the autonomic nervous system (ANS) are observed in many mental disorders, including addictive disorders (20, 21). Heart rate variability (HRV), which is extracted through electroencephalogram (ECG) data, is one of the important bio-signals that reflect the responsiveness of an individual’s ANS (22). HRV reflects not only ANS functioning but also emotional state and cognitive control, and has been widely used in the investigations of psychiatric problems (23, 24). Resting-state HRV was used as a bio-signal related to the clinical aspects of addiction diseases (25). There were previous studies that analyzed resting-state HRV features in IGD, and they reported that the high frequency (HF)-HRV index, which reflects prefrontal control, was reduced in subjects with IGD (26). Notably, HRV can be applied to wearable devices and has the advantage of being able to be measured not only in a resting state but also in an active state (27). Measuring changes in HRV according to changes in certain conditions is useful in that it can reflect autonomic reactivity to certain stimuli (28). Some previous studies have used HRV measurement to evaluate cue-reactivity in addiction diseases (29, 30). Previously, we measured HRV for gaming situations and found that IGD subjects’ HF-HRV during game play was significantly lower than HF-HRV during resting state or attentional task performance (31, 32). These previous studies measured the HRV changes of IGD subjects to actual game play of their addicted game. This increased the possibility that subjects perceived it as a salient stimulus compared to the static gaming-related cues (game-related words or pictures) presented in the experimental environment. However, our previous studies only performed dichotomous comparisons between subjects with IGD and healthy controls. We did not explore whether HRV parameters are useful bio-signals in predicting the severity of IGD.

In this study, we implemented a deep learning model, which predicts the severity classification of IGD, using HRV reactivity to gaming cues as an input. A deep learning model is one of the machine learning algorithms that self-learns from data using artificial neural networks (33). It performs feature extraction and classification on its own based on the data rather than on existing knowledge and experience. Psychiatric disorders generally have heterogeneous clinical and neurobiological characteristics, and the relationship between mental disorders and neurobiological pathophysiology has not been established in many cases, so the usefulness of psychiatric disorder classification through deep learning models is attracting attention (34). Deep learning models have been mainly used to classify psychiatric disorders through neuroimaging findings (35), but some studies have also been conducted on HRV data (36). There were previous studies that applied deep learning models to IGD (37, 38), but no studies using HRV data. Using a deep learning model, the usefulness of HRV in assessing IGD can be explored, and which HRV parameters are extracted for classifying IGD can be explored.

We tried to identify the following through this study. First, this study aimed to verify whether HRV reactivity in response to a gaming situation could reflect the severity of IGD. Young adults immersed in the Internet game were evaluated through a self-report questionnaire to classify the degree of IGD severity. We explored whether HRV’s cue-reactivity for actual gameplay can predict severity classification of IGD through a deep-learning model. Second, we tried to identify specific HRV parameters related to IGD. Among HRV parameters, it has been found that HF-HRV is specifically related to parasympathetic tone and low frequency (LF)-HRV is related to sympathetic tone (39). In this study, we explored which HRV parameters were extracted as features in the deep learning model predicting the severity classification of IGD.

## Methods

### Participants

This study was conducted on 70 male adults (mean age: 22.0 ± 2.8 years). The age group from 19 to 29 years old was included. Considering that there are more males in IGD (40), this study only targeted male subjects in order to minimize gender differences. Subjects were recruited from those who were immersed in Internet games via the online bulletin board. This study was conducted in Seoul, Korea, one of the metropolis. All screenings in this study were conducted through face-to-face interviews by certified psychiatrists. In order to reduce the influence of individual preference on the gaming stimuli, all participants were recruited from those who frequently play the online game, “League of Legends (Riot Games, 2009).” In order to minimize the difference in familiarity, subjects were limited to those who are ranked at a similar level in the game (Gold or Silver Level). Those with a history of major psychiatric disease (e.g., major depressive disorder, bipolar disorder, psychotic disorder, alcohol use disorder) or a history of taking psychiatric medications were excluded. All subjects were evaluated through the Korean version of the Structured Clinical Interview for DSM-IV-TR (SCID-IV) and confirmed to have no major psychiatric problems (41). Subjects with limited linguistic or cognitive function to understand the research process were excluded. Intelligence (IQ) was also evaluated using the Korean version of the Wechsler Adult Intelligence Scale IV (WAIS-IV) (42). Those with an IQ of less than 80 were excluded. Ethics approval was obtained from the Institutional Review Board of Hanyang University (HYI-16-044).

After the evaluation through face-to-face interviews, several questionnaire evaluations were conducted through the paper. After informing the subjects that the online activity to be covered in the questionnaire in this study was the use of Internet games, the Young Internet addiction test (IAT) was conducted to assess the severity of IGD (43). The IAT is a self-reported questionnaire that evaluates the addictive state of online activities. It is a 5-point Likert scale consisting of 20 questions and scored from 20 to 100 points. The IAT has the advantage of being simple and easy to use, and has been frequently used in IGD research (44). The IAT consists of items that can evaluate the preoccupation with the Internet (e.g., “Do you choose to spend more time online over going out with others?”) and items that can evaluate the loss of control and the interference with daily life (e.g., Do you feel that you stay online longer than you intend?”) (45). We classified the subjects into 4 classes according to their IAT scores: ‘Normal’ with a score of 0–30, ‘Mild’ with a score of 31–50, ‘Moderate’ with a score of 51–80 and “Severe” with a score of 81–100 (46). There were 15 subjects in the normal class, 30 subjects in the mild class, 23 subjects in the moderate class and 2 subjects in the severe class.

In addition to the IAT, several self-report questionnaires were conducted to evaluate the subjects. We used the Beck depression inventory (BDI) for depression (47), the Beck anxiety inventory (BAI) for anxiety (48), the Barratt impulsiveness scale (BIS) for impulsivity (49), and the alcohol use identification test (AUDIT) for alcohol use problems (50). Because of the high association between IGD and attention deficit hyperactivity disorder (ADHD) (51), a questionnaire evaluation of ADHD was also conducted. The Conners ADHD rating scale (CAARS) was used to assess symptoms of ADHD (52). In addition, the Wender Utah rating scale (WURS) was used to evaluate the ADHD symptoms of the subjects during their childhood and adolescence (53).

### Experiments

Gameplay and HRV measurement in this study were conducted in an independent research space in a university building. Although the research space was not a space where subjects normally played games, a laptop and related equipment were set up so that there were no restrictions on gameplay. In this study, Ag-AgCl electrodes were placed on the subject’s torso (thorax) to minimize noise generated during the game. Electrodes were attached according to the Eindhoven triangle (54). Three ECG channels were connected to electrodes to obtain ECG signals through the MP150 (BIOPAC Systems Inc., Santa Barbara, CA, United States). The electrocardiogram (ECG) data were acquired in a resting state for 5 min before the gameplay. Afterwards, subjects played their favorite online game (League of Legends) three times. In order to induce appropriate immersion in the experiment, subjects conducted the experiment using game episodes that affect their online game level, and the gameplay was allowed to last for at least 20 min. ECG data while the subjects were playing the game was acquired.

### HRV preprocessing

In the preprocessing step, we used 3rd-order Butterworth high-pass filter with 1 Hz cutoff frequency to remove baseline noise caused by movement or respiration of the subject. A 3rd-order Butterworth lowpass filter with 15 Hz cutoff frequency was used to remove noise caused by muscle contraction and movement. We also used a 6th order Butterworth notch filter to remove 60 Hz power supply noise. Detecting consecutive R-Peak was applied using the Pan & Tompkins algorithm (55). In this study, the polynomial detrending method was used to remove the ectopic beat by using a 20% filter and to remove the low frequency tendency of the HRV signal. In order to minimize the influence of the high-frequency signal on the power spectrum, the inter-bear interval (IBI) was cubically interpolated at 4 Hz.

The frequency domain analysis method of the HRV can only obtain information on how the power of the R-Peak signal is distributed in the frequency domain. This method does not provide spectrum information according to time variation. On the contrary, the time-frequency domain analysis method provides spectrum information according to time variation. The time-frequency domain analysis quantifies VLF (0 ~ 0.04 Hz), LF (0.04 ~ 0.15 Hz), and HF (0.15 ~ 0.4 Hz) parameters. In this study, windowed Fourier transform (short-time Fourier transform) and continuous wavelet transform with a 30 s window length and 20 s overlap size were used for the time-frequency domain analysis.

### HRV analysis and deep learning modeling

First, HRV was analyzed for ECG data during a 5-min rest period before playing the game (“baseline HRV”). To reflect HRV reactivity for gaming situation, we analyzed HRV values extracted from ECG data for 5 min after the start of each game episode (“gameplay HRV”). Since each subject had 3 game episodes, the HRV analysis of each subject was targeted for a total of 900 s. In order to construct a 30-s data set from 300-s HRV data, shifts were performed 28 times every 10 s. Taken together, a total dataset of 5,880 EA (70 Subjects X 3 times game X 28 times shift) was constructed.

HRV signal changed to the time-frequency domain was composed of 304 frequency bins with a frequency component of 0 to 0.4 Hz and 30 times bins with a time of 30 s. By using these signals as input (size: 304 × 30 × 1), we trained the deep learning model. To minimize the influences of individual factors (i.e., cardiovascular condition, physical activity level and body mass index), the “gameplay HRV” was normalized based on the “baseline HRV” of each subject. In the normalization, the value obtained by averaging power spectral density (PSD) of each frequency bin of “baseline HRV” was used.

In this study, the deep learning model was based on VGG16 trained on a subset of the ImageNet database. The 2D convolution neural network was constructed for IGD severity prediction. The input of the deep learning model was composed of 9,120 EA (304 Frequency bin X 30 Times X 1 Power). The output of the deep learning model was 4 classes (Normal, Mild, Moderate and Severe). We performed the 10-fold validation to prevent the overfitting. Data from subjects who did not belong to the training set were utilized for the test set. In the Class Activation Mapping (CAM) method, if the convolution filter of the learned deep learning model is reversed and the activated position is traced, the region of interest of the deep learning model for the input information can be found (56). Using this method, we analyzed the area of interest for the input information of the deep learning model predicting IGD severity.

### Statistical analysis

For statistical analysis other than deep learning modeling, the Statistical Package for the Social Sciences (SPSS) version 24.0 (IBM Corp., Armonk, NY, United States) was used. Statistical significance was determined based on a *p* value <0.05. The clinical variables of the subjects between classes were compared through one-way analysis of variances (ANOVA). Since the number of subjects in the severe class was too small, comparison was made between the remaining 3 classes.

## Results

### Clinical characteristics of the subjects

Comparison of the demographic and clinical characteristics of the subjects was made within 3 groups except for the severe group because the number of subjects in the severe group was too small (Table 1). There was a significant difference between groups in the age of the subjects, and the mild group was older than the normal group. There was no significant difference between groups for FSIQ. Regarding impulsivity, the moderate group showed higher impulsivity than the mild and normal groups. Other self-report questionnaires (BDI, BAI, CAARS, and WURS) did not show significant differences between groups.

**Table 1:** Demographic and clinical characteristics of subjects.

	Normal (*n* = 15)	Mild (*n* = 30)	Moderate (*n* = 23)	Severe (*n* = 2)	*F*-test^a^	*p*-value^b^	Comparisons
Age, years	20.7 ± 2.0	22.9 ± 3.1	22.2 ± 2.8	24.5 ± 0.7	3.256	0.045	Mild > Normal
Young internet addiction test	19.6 ± 8.5	38.7 ± 5.6	61.1 ± 8.2	81.0 ± 1.4	155.548	<0.001	Moderate > Mild > Normal
Full scale intelligence quotient	108.5 ± 11.3	114.0 ± 11.3	110.2 ± 14.5	115.5 ± 10.6	1.158	0.320	
Beck depression inventory	6.0 ± 4.1	7.6 ± 5.2	10.2 ± 9.6	3.5 ± 2.1	1.839	0.167	
Beck anxiety inventory	4.1 ± 3.0	5.9 ± 6.3	5.9 ± 4.8	1.5 ± 0.7	0.650	0.525	
Barratt impulsiveness scale	47.2 ± 5.1	50.5 ± 6.6	56.4 ± 8.1	54.5 ± 5.0	8.969	<0.001	Moderate > Normal, Mild
Alcohol use disorder identification test	10.2 ± 6.0	9.3 ± 5.4	11.1 ± 7.6	6.5 ± 2.1	0.524	0.595	
Conners ADHD rating scale							
Inattention/memory problems	48.5 ± 4.4	50.9 ± 4.3	51.2 ± 5.2	52.5 ± 7.8	1.814	0.171	
Hyperactive/restlessness	45.8 ± 3.6	47.9 ± 4.6	48.8 ± 5.2	49.5 ± 0.7	1.977	0.147	
Impulsive/emotion lability	46.5 ± 2.8	48.8 ± 4.5	48.4 ± 4.1	46.5 ± 3.5	1.555	0.219	
Problems with self-concept	59.5 ± 6.7	59.3 ± 7.7	65.2 ± 11.9	60.5 ± 9.2	3.129	0.050	
Wender Utah rating scale	25.8 ± 15.4	23.7 ± 13.7	25.7 ± 15.8	31.5 ± 30.4	0.160	0.852	

Values were presented by mean ± SD; SD standard deviation. ADHD: attention deficit hyperactivity disorder. ^a, b^The one-way analysis of variances (ANOVA) were conducted within 3 groups except for the severe group.

### Deep learning model

**Figure 1:**
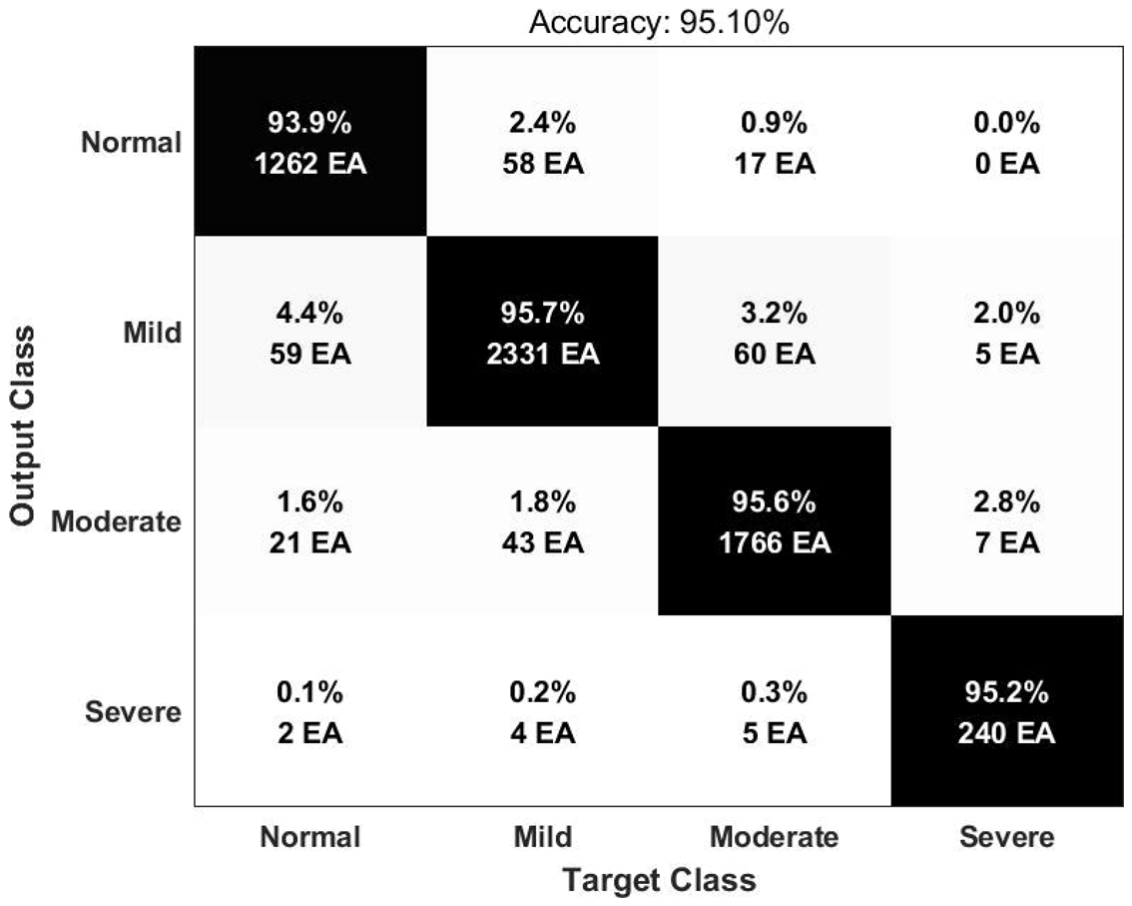
Confusion matrix for deep learning model in this study.

Deep learning input is composed of two-dimensional image by expressing the time-frequency domain analysis result of HRV as Scalogram. In order to optimize the deep learning structure, a convolutional layer was constructed based on the VGG16 model. We checked the confusion matrix and receiver operating characteristic (ROC) curves for performance evaluation. The result of the accuracy for classification of 4 classes (Normal, Mild, Moderate, and Severe) was 95.10% (Figure 1). The area under the ROC curve (AUC) was 0.9951 for the normal group, 0.9941 for the mild group, 0.9957 for the moderate group and 0.9992 for the severe group.

**Figure 2:**
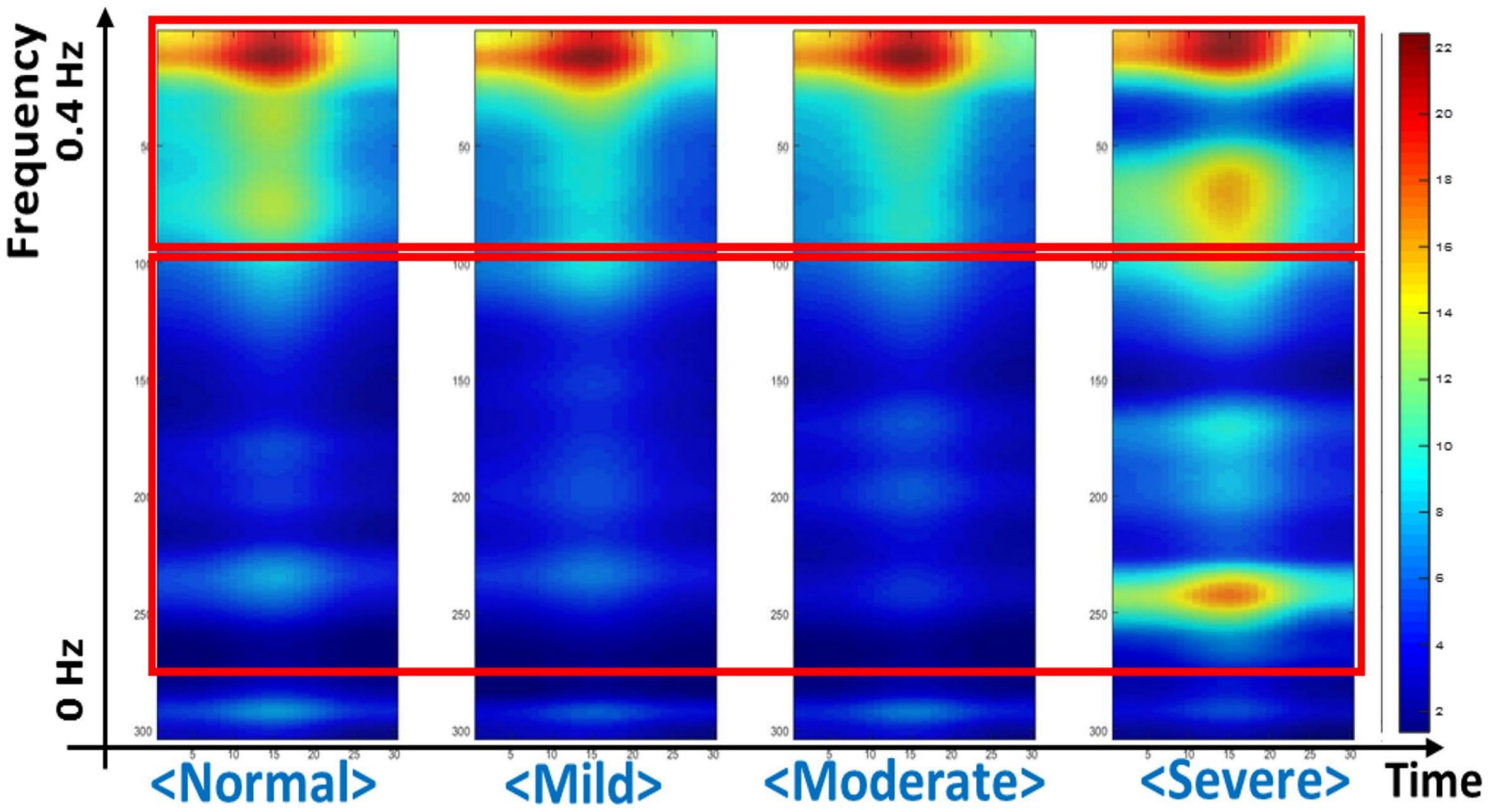
Regions of interest referenced in the deep learning model, identified through the class activation mapping.

In this study, the CAM method was used to explore which area was referred to in the deep learning model for classification according to IGD severity (Figure 2). As a result, the deep learning model referred the HF parameters as the regions of interest for classification. In the severe group, both of the HF and LF parameters were used in the deep learning model as the regions of interest.

## Discussion

In this study, a deep learning model with HRV time-frequency parameters was used to predict groups according to IGD severity. As a result, the deep learning model using HRV parameters was able to reliably predict classes according to the severity of IGD. The results of this study are consistent with previous studies that reported alterations of ANS in IGD (26). In addition, this study suggested that HRV may have clinical value in IGD as well as in other addiction diseases (57). Notably, the HRV parameters used in the deep learning model of this study were measured when exposed to actual gameplay situations. These HRV parameters acquired during gaming was normalized based on baseline resting HRV in order to reflect the reactivity of HRV for actual gaming situations. Therefore, the results of this study suggest that the autonomic response to gaming situations may reflect the state of addiction to games. Our findings suggest that, in IGD as in other behavioral addictive diseases (17), changes in cue-reactivity to addiction-related stimuli may be one of the crucial pathophysiological factors. Moreover, this study was able to reflect gaming-specific cue-reactivity, since we measured HRV while subjects were playing a game in which they were actually immersed.

We explored which HRV parameter area was mainly referred to by our deep learning model when predicting the severity of IGD. As a result, it was found that the region corresponding to HF-HRV was mainly referred to predict IGD severity. HF-HRV, which is usually known to reflect parasympathetic tone, has been found to show a significant correlation with executive control (58). In the IGD’s disease model, executive control dysfunction has been suggested as a major mechanism for losing control over excessive game use (9). Therefore, our current results support that executive control dysfunction is an underlying factor that can reflect the clinical features of IGD. In particular, considering that this study measured HRV reactivity for the gaming situation, our current findings suggest that the weakening of executive control in response to facing a gaming cue is related to IGD’s pathophysiology. Our current findings are consistent with integrative models of addictive behaviors that proposed stimuli-specific reduction in executive control (18).

In this study, in the case of the severe group, not only the HF-HRV area but also the LF-HRV area were referenced in the deep learning model. The LF-HRV has been found that not only parasympathetic but also sympathetic tone is innervated (59). A recent study of bio-signals acquisition in IGD found an association between craving for gaming in IGD subjects and high levels of sympathetic arousal for gaming-related stimuli (60). Therefore, the results of this study suggest that autonomic arousal for gaming-related stimuli is related to severe level of IGD. In addition, LF-HRV has been found to reflect the baroreceptor reflex functioning (61), and alterations in the baroreceptor reflex were identified as one of the biological responses to chronic stress reactions (62). Therefore, the results of this study are consistent with the speculation that high stress vulnerability is linked to the predisposition to IGD (63). However, careful consideration should be taken in interpretation because the number of subjects in the severe group was too small and meaning of LF-HRV is controversial (64). Further studies involving a larger number of subjects and other indicators for autonomic arousal or stress reaction would be needed to verify the current findings.

This study had several limitations. First, the number of subjects was not sufficient. In particular, as mentioned above, there were very few subjects in the severe group. It is necessary for future studies to include a larger number of subjects for each group. Second, this study included only male subjects. Although there are generally more males than females in IGD, it has recently been revealed that female IGD patients are not uncommon and that they may show different clinical characteristics from males (65). Therefore, inclusion of both genders will help verify and generalize the results of this study. Third, in the evaluation of IGD, this study did not use an evaluation tool specific to Internet gaming, but instead used the scale for overall online activity. However, this study recruited subjects who were skilled and familiar with the same game (‘League of Legends’). In addition, the subjects were reminded that the Internet-related activity, which is the main target of the IAT questionnaire in this study, was Internet gaming. Fourth, this study did not fully explore the psychological profiles of the subjects. Addictive behavior is closely related to various psychological characteristics such as personality traits, behavioral motives, and stress coping (66, 67). If these psychological characteristics were comprehensively explored, deeper insights could be given in exploring the relationship between addictive gaming behavior and HRV features. Fifth, this study utilized only the HRV data of the first 5 min of the game. The reason for focusing only on HRV data at the beginning of the game was to be less affected by the progress of the game, and also to explore the autonomic reaction after a gaming-related cue was given. However, if a wider range of HRV data were analyzed more extensively, a richer interpretation of the ANS alterations of IGD could have been derived. Sixth, this study did not sufficiently control physiological variables that could affect HRV. Respiration can have a significant impact on HRV, for example, deep and slow breathing can increase the power of HF-HRV (68). HRV analysis that measures changes in respiration along with heart rate and precisely controls the influence of breathing will be needed in future research on HRV during gameplay.

Despite the above limitations, this study suggested that HRV reactivity to gaming-related stimuli could reflect the clinical condition of IGD. Recently, there are various type of wearable devices that can measure heart rate have been released. Therefore, the results of this study support that measuring bio-signals during actual game play through a wearable device could have clinical implications in the evaluation of IGD. This study also warrants further research on whether HRV monitoring is meaningful for the assessment of behavioral addictions other than IGD (69, 70). In addition, this study showed that HF-HRV, which reflects parasympathetic tone, was significantly referenced in the deep learning model for IGD. This finding suggests that the weakening of executive control in response to gaming-related stimuli is associated with the pathophysiology of IGD.

## Data availability statement

The raw data supporting the conclusions of this article will be made available by the authors, without undue reservation.

## Ethics statement

The studies involving humans were approved by the Institutional Review Board of Hanyang University (HYI-16-044). The studies were conducted in accordance with the local legislation and institutional requirements. The participants provided their written informed consent to participate in this study.

## Author contributions

Y-MS and IYK contributed to the study design. JP and TK collected the clinical and bio-signal data. SJH and DL performed statistical analysis and wrote the first draft of the manuscript. Y-CJ and IYK provided critical revision of the manuscript and important intellectual content. All authors contributed to the article and approved the submitted version.
